# Molecular insights into regulatory RNAs in the cellular machinery

**DOI:** 10.1038/s12276-024-01239-6

**Published:** 2024-06-14

**Authors:** Sumin Yang, Sung-Hyun Kim, Eunjeong Yang, Mingon Kang, Jae-Yeol Joo

**Affiliations:** 1 Department of Pharmacy, College of Pharmacy, Hanyang University, Ansan, Gyeonggi-do 15588 Republic of Korea; 2 Department of Computer Science, University of Nevada, Las Vegas, NV 89154 USA

**Keywords:** Gene regulation, Long non-coding RNAs

## Abstract

It is apparent that various functional units within the cellular machinery are derived from RNAs. The evolution of sequencing techniques has resulted in significant insights into approaches for transcriptome studies. Organisms utilize RNA to govern cellular systems, and a heterogeneous class of RNAs is involved in regulatory functions. In particular, regulatory RNAs are increasingly recognized to participate in intricately functioning machinery across almost all levels of biological systems. These systems include those mediating chromatin arrangement, transcription, suborganelle stabilization, and posttranscriptional modifications. Any class of RNA exhibiting regulatory activity can be termed a class of regulatory RNA and is typically represented by noncoding RNAs, which constitute a substantial portion of the genome. These RNAs function based on the principle of structural changes through *cis* and/or *trans* regulation to facilitate mutual RNA‒RNA, RNA‒DNA, and RNA‒protein interactions. It has not been clearly elucidated whether regulatory RNAs identified through deep sequencing actually function in the anticipated mechanisms. This review addresses the dominant properties of regulatory RNAs at various layers of the cellular machinery and covers regulatory activities, structural dynamics, modifications, associated molecules, and further challenges related to therapeutics and deep learning.

## RNA’s role in cellular machinery: insights from transcriptome studies

Regulatory RNAs, such as long noncoding RNAs (lncRNAs, RNAs that do not code for proteins), microRNAs (miRNAs, small RNAs that regulate gene expression), and circular RNAs (circRNAs, RNAs that form a covalently closed continuous loop), are important in controlling gene expression. The exact ways and roles of these RNAs are not completely known. This study by Joo et al. reviews current knowledge on regulatory RNAs, focusing on their structure and function in cell parts. The authors talk about the different methods used to study these RNAs, including RNA-Chromatin, RNA-Protein, and RNA structure sequencing. They also emphasize the role of RNA modifications in controlling gene expression and the potential of deep learning (a type of machine learning) in predicting RNA functions. The study concludes that understanding regulatory RNAs better could lead to new treatment strategies for various diseases.

This summary was initially drafted using artificial intelligence, then revised and fact-checked by the author.

## Introduction

Regulatory RNAs exhibit highly dynamic properties due to their multidimensional structures and their ability to interact with RNA, DNA, and proteins. These RNAs are involved in regulated events, such as transcription, translation, molecular localization and stability, and enzymatic degradation cascades, on many levels^1–4^. A broad spectrum of RNAs that have regulatory functions are collectively recognized as regulatory RNAs. Historically, many tiers of noncoding RNAs (ncRNAs) have been identified that have diverse characteristics. These ncRNAs account for approximately 98% of the total transcriptome, and while some ncRNAs exhibit protein-coding ability^5^, the majority of ncRNAs are expected to function as regulatory RNAs^6,7^. Regulatory RNAs can be classified into different groups based on their size, structure, localization, and function, as indicated by the discovery of long noncoding RNAs (lncRNAs), enhancer RNAs (eRNAs), microRNAs (miRNAs), PIWI-interacting RNAs (piRNAs), small Cajal body-specific RNAs (scaRNAs), small nucleolar RNAs (snoRNAs), small nuclear RNAs (snRNAs), and circular RNAs (circRNAs)^7,8^. The enormous functional involvement of regulatory RNAs—despite their low expression level and low sequence conservation—is undeniable, and they apparently play important roles in cellular phenomena through these functions^9^. Research on RNAs and their biogenesis has described both shared and distinctive features among the numerous types of regulatory RNAs. Numerous regulatory RNAs undergo mRNA-like RNA processing steps, such as 5′ m^7^G cap addition, polyadenylation, and splicing, which are observed even in long RNAs. The pattern of overlapping transcripts in the genome indicates the inclusion or exclusion of exons and introns in the sequences of regulatory RNAs, which influences the open reading frame^10^. Additionally, the directionality of transcription is can be either unidirectional or bidirectional, possibly implying base pairing-dependent functions^11^. LncRNAs, with more than 200 nucleotides, are the most common type of RNA undergoing mRNA-like processes, and the lncRNA class contains numerous kinds of regulatory RNAs due to the arbitrary definition of this category considering only length. Numerous lncRNAs are involved in multiple mechanisms in multiple cellular components. They interact with other RNA molecules or proteins, thereby modulating cellular signaling and epigenetic regulation^12–15^. Active enhancer sequence-derived eRNAs are considerably lengthy, i.e., more than 1 kilobase, and are responsible for enhancer-associated contributions^16,17^. Given this knowledge, miRNAs are also commonly referenced regulatory RNA class. The biogenesis of regulatory miRNAs involves several sequential steps. A hairpin-structured primary miRNA (pri-miRNA) is cleaved by the RNase III Drosha to a length of 55–70 nucleotides in the nucleus; the resulting pre-miRNA is released into the cytoplasm and is then cleaved into a miRNA duplex by the RNase III Dicer. Mature miRNAs bind to complementary mRNAs, repressing translation through an interaction in the 3′ untranslated region (UTR) of the target mRNA. The RNA-induced silencing complex (RISC) and argonaute cooperate to actively regulate target interference^18–20^. Similar to miRNAs, piRNAs utilize argonaute but are distinguished by their sequences, mainly derived from transposons, and are comparatively more involved in epigenetic regulatory processes^21^. Regulatory RNAs found in nucleoli, termed snoRNAs, have a unique set of sequences that allow their subclassification into C/D box snoRNAs (SNORDs), H/ACA box snoRNAs (SNORAs), and far more specific localizing motifs define scaRNAs found only in Cajal bodies. These motifs lead to 2′-O-methylation and pseudouridylation modifications in other classes of RNA, conferring regulatory identity^22,23^. Another unique class, circRNAs, possess a closed circular structure formed by back-splicing of pre-mRNAs, unlike the linear form of other regulatory RNAs. Their splicing determines the location of their function; exonic circRNAs are transported to the cytoplasm, whereas intronic and intro-exon circRNAs remain in the nucleus^24,25^.

Currently, research is at an intersection defined by exponentially increasing numbers of datasets generated through high-throughput sequencing techniques^26^. This surge is essential for genomic research, especially in defining the pool of regulatory RNAs. For instance, RNA sequencing and chromatin immunoprecipitation sequencing aided in enabling eRNA discovery and investigation^27,28^. Multiple kinds of sequencing methods have been adopted to examine regulatory RNAs in detail with procedural commonalities, from RNA preparation to analysis of aligned sequences (Table 1). Numerous efforts have been made to determine the regulatory functions of RNAs^29,30^. The interaction of regulatory RNAs with accompanying molecules has been a key factor in designing sequencing methods. RNA-associated chromosomal conformation studies can be conducted with techniques such as Hi-C coupled with chromatin isolation by RNA purification, chromatin-associated RNA sequencing, and RNA and DNA interacting complexes ligated and sequenced^31–34^. To identify proteins that specifically bind to RNA, methods such as UV crosslinking with immunoprecipitation^2,35,36^, photoactivatable ribonucleoside-enhanced crosslinking and immunoprecipitation^2,37,38^, and individual-nucleotide resolution UV crosslinking and immunoprecipitation can be adapted^2,39,40^. As alterations in RNA structure directly affect RNA function, sequencing methods such as RNA in situ conformation sequencing (RIC-seq)^41,42^, mapping RNA interactome in vivo^43^, and dimethyl sulfate mutational profiling with sequencing^44–46^ can be utilized to investigate such RNA structure-dependent aspects. Additionally, the RNA:DNA hybridization-mediated structure and its attributes can be confirmed by DNA‒RNA immunoprecipitation coupled with high-throughput sequencing (DRIP-seq)^47–49^, DNA:RNA immunoprecipitation followed by cDNA conversion coupled with high-throughput sequencing (DRIPc-seq)^47,50^ and bisulfite-based DRIP-seq^51^. More specifically, single-molecule structure sequencing can be used to investigate tertiary interactions, the dynamics of riboswitch ligand binding, and mRNA structural features^1^. With respect to the subcellular localization of regulatory RNAs, spatial transcripts perform distinct functions across tissues according to the spatial context. Advanced single-cell sequencing techniques allow researchers to access highly resolved spatial transcriptomes at intercellular resolution without external perturbation^52–54^. Databases focusing on regulatory RNAs, such as lncRNAdb, Rfam, miRBase, starBase/ENCORI, and NONCODE, have been improved to support annotation and network analysis^55–59^.

**Table 1:** Sequencing methods for regulatory RNA analysis.

Method	Description	Property	Yield	Ref.
*RNA-Chromatin*				
HiChIRP	-RNA pulldown using biotinylated probes modified based on the ChIRP method	-Cannot be used to investigate chromatin interactions with unknown RNAs	300–700 bp DNA	^32,33^
ChAR	-Proximity ligation of RNA-chromatin contacts to identify RNA‒DNA interactions -Mapping of the locations of RNAs on the genome -Dpn II restriction enzyme is limited to detecting RNA‒DNA interactions	-Reduced nonspecific interactions	20–100 bp RNA, DNA	^33,34^
RADICL	-Proximity ligation-based enhanced genome coverage allows the discernment of unique RNA-chromatin interaction patterns	-Reduced unintended interactions by in situ ligation within intact nuclei	225 bp RNA‒DNA complex	^34^
*RNA‒Proteins*				
CLIP	-Identification of RNA-specific protein interactions by UV crosslinking and immunoprecipitation -Technical bias due to crosslinking efficiency and antibody specificity	-Detection of all RNAs interacting with a specific protein of interest	70–100 nt RNA	^2,35,36^
PAR-CLIP		-Enhanced crosslinking efficiency by incorporating photoactivatable nucleosides analogs	20–40 nt RNA	^2,37,38^
iCLIP		-Different cDNA protocol compared to CLIP, PAR-CLIP -Labeling of individual cDNAs with stochastic barcodes to minimize quantitative bias	50–300 nt RNA	^2,39,40^
*RNA structure*				
RIC	-Enables the capture of secondary and tertiary interactions within RNAs	-Targets noncoding RNA counterparts and distinguishes topological domains in trans-interactions for RNA molecules	90 nt RNA 100 nt C–biotin-RNAs	^41,42^
MARIO	-Uses a biotinylated RNA linker to enable the mapping of endogenous RNA interactions	-Uncovers all interactions among RNA molecules	150–350 bp DNA	^43^
DMS-MaP	-Dimethyl sulfate modification of single-stranded regions in RNA allows the identification of modified sequences and the inference of RNA secondary structures	-Reveals RNA secondary structures at single-nucleotide resolution	150 nt RNA	^44–46^
SMS	-Enables structural profiling of individual RNAs without amplification -Ability to identify long-range interactions heavily relies on a substantial modification rate coupled with optimal RNA integrity	-Identifies tertiary interactions, including riboswitch ligand binding and the structural characteristics of mRNAs	−	^1^
*RNA:DNA hybridization (R-loop)*				
DRIP	-Uses the S9.6 antibody against RNA:DNA hybrids to capture the structure -Reveals precise epigenomic patterns related to R-loop formation -Sensitive to RNase H pretreatment	-Limited resolution and information on strand specificity	150–500 bp DNA	^47–49^
DRIPc		-Increased strand specificity compared to DRIP -DRIP followed by cDNA conversion -Requires a large amount of DNA (30-40 µg) compared to DRIP	200–500 bp DNA	^47,50^
bisDRIP		-DRIP following bisulfite-induced cytosine-to-uracil conversion -Low sensitivity	~300 bp DNA	^51^

Research on the roles of regulatory RNAs throughout the cell has accelerated. Studies incorporating advanced deep sequencing techniques have provided important insights through measurements of RNA structure, dynamics, and affinity. Guided by base pairing, regulatory RNAs have physiological functions in development, the cell cycle, and disease processes, influencing various biochemical pathways within the cell. A plausible complete sequence of each chromosome has only recently become accessible through long-read sequencing. Therefore, most of the functions of regulatory RNAs remain unclear^60^. In this review, we explore the current knowledge of regulatory RNAs, focusing on their structural dynamics and function in cellular components. We discuss nucleobase modification and protein cooperation in cellular mechanisms and, at the end of the review, we present RNA-based clinical approaches and ideas for using deep learning techniques.

## RNA dynamics-based regulatory functions

**Fig. 1:**
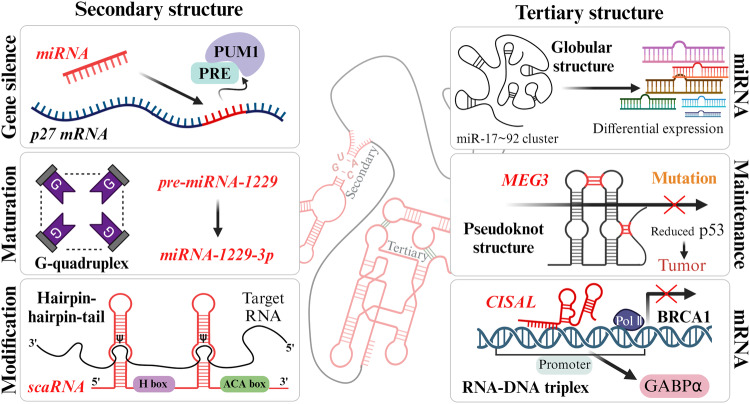
The secondary and tertiary structures mediate the various functions of regulatory RNAs. The dynamic structure of RNA is simply classified as the secondary a tertiary structures formed by specific base pairing and numerous factors, and various RNAs affect biological functions via these structures. In regulatory RNAs, the secondary structure is related to gene silencing via miRNA expression, miRNA maturation, and RNA modifications via scaRNAs. The tertiary structure participates in the regulation of p53 and the differential expression of miRNAs and mRNAs.

The activities and functions of RNAs are influenced by their secondary or tertiary structures, which can be altered through various factors involving the binding of DNA, other RNAs, metal ions, proteins and metabolites, posttranscriptional modifications, mutations, and alterations in environmental conditions (Fig. 1). In recent years, multifaceted analytical approaches have been used to demonstrate correlations between RNA conformational dynamics and cellular functions, including the regulation of gene expression, alternative splicing, ribonucleoprotein assembly, and miRNA maturation. Thus, a comprehensive understanding of these structures can contribute to elucidating their functional mechanisms^61,62^.

The secondary structure of an RNA is formed by interactions between specific nonadjacent bases in the primary nucleotide sequence. The four canonical RNA bases, adenine (A), uracil (U), guanine (G), and cytosine (C), are paired through hydrogen bonds according to the Watson-Crick principle (A-U and G-C), and wobble base pairing between G and U often occurs. These hydrogen bond-based base pairs contribute significantly to the stabilization and function of RNA and result in several secondary structure conformations, such as hairpin loops, bulge loops, inner loops, multibranched loops, single-stranded regions, helices and pseudoknots^63–65^. Gene silencing is a type of posttranscriptional process mediated by an RNA switch caused by miRNA binding. For instance, the 3′ UTR of p27 mRNA serves as the region where this RNA switch regulates mRNA expression. This involves the simultaneous release of the Pumilio-recognition element (PRE) from the Pumilio RNA-binding family member 1 (PUM1) cofactor, allowing miRNA binding. The interaction between PRE and PUM1 initiates the formation of the secondary structure that exposes the target site of the mRNA, promoting miRNA-mediated silencing^66^. Pre-miRNA-1229 is processed into mature miR-1229-3p through a noncanonical secondary structure, namely, a G-quadruplex that is balanced by canonical hairpin loops. In Alzheimer’s disease (AD), pre-miRNA-1229 containing the SNP rs2291418 induces structural malformations associated with pathological progression^67^. On the other hand, the scaRNA is the specific guide RNA for 2′-O-methylation (2′-O-Me) and pseudouridylation (ψ), which are associated with posttranscriptional modification of snRNA maturation in spliceosomal small nuclear RNPs (snRNPs). This RNA forms a “hairpin–hinge–hairpin–tail” secondary structure with the target snRNA through conserved H/ACA box elements, contributing to ψ via specific factors^68,69^.

Tertiary structures are three-dimensional RNA building blocks resulting from the assembly of two or more secondary structures. These conformations are mediated via base stacking, noncanonical base pairing, the formation of triplex structures involving base triples, metal ions, metabolites, and interactions between unpaired bases and the ribose–phosphate backbone. The interactions between secondary structures and cofactors consequently lead to the formation of higher-order tertiary RNA structures with specific functions, such as scaffolding, regulation, and catalysis^61,70^. One cluster of pri-miRNAs contains six putative miRNA sequences: miR-17, miR-18a, miR-19a, miR-20a, miR-19b-1, and miR-92a. These six pri-miRNAs show different efficiencies for pre-miRNA generation in an RNA tertiary structure-dependent manner, as distal miRNAs are released more efficiently than internalized core proximal miRNAs in a globular tertiary structure. This three-dimensional conformation-specific expression of miRNAs from the miRNA cluster miR-17–92 is closely related to development and tumorigenesis^71–73^. Additionally, the human maternally expressed gene 3 (*MEG3*) lncRNA is composed of two highly conserved distal motifs, which are connected via complementary base pairing as a pseudoknot structure (kissing loops). *MEG3* regulates the p53 pathway as a tumor suppressor, while mutated *MEG3* inhibits the formation of the pseudoknot, leading to a reduction in the p53 response^74^. The lncRNA *LINC01011*, a cisplatin sensitivity-associated lncRNA *(CISAL)*, regulates mitochondrial fission in tongue squamous cell carcinoma. *CISAL* forms an RNA‒DNA triplex structure by binding directly to the promoter of breast cancer 1 *(BRCA1)*, which induces the dissociation of GA-binding protein transcription factor subunit alpha (GABPα), the transcription factor for *BRCA1*^75^.

Modification of RNA structures has consistently been implicated in numerous diseases, including heart failure and neurological disorders, such as AD. However, these dynamic conformations may form within picoseconds, while the formation of others may take seconds, with various factors modifying RNA structures intracellularly and extracellularly^76–78^. Therefore, further studies are needed to elucidate the background of RNA structure-specific cellular functions under multiple conditions.

## Regulatory RNAs in cellular components

**Fig. 2:**
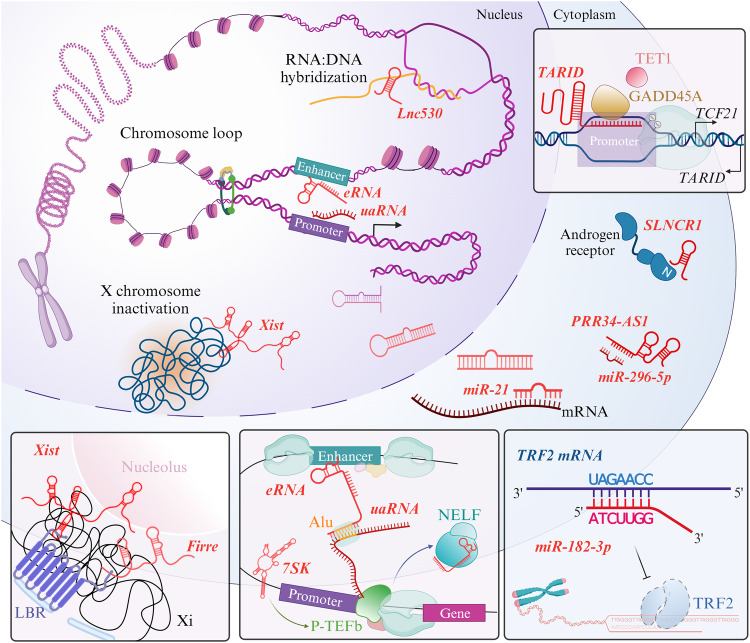
Versatile functions of regulatory RNAs in the subcellular molecular machinery. Physiological interactions of regulatory RNAs with the cellular machinery occur in both the nucleus and cytoplasm. In the nucleus, chromosome looping facilitates enhancer and promoter interactions to regulate gene expression; here, enriched eRNAs on active enhancer regions and uaRNAs near promoters lead to selective binding. In RNA:DNA hybridization, R-loops can be formed by mRNAs and ncRNAs to regulate gene expression. Inactivated X chromosomes are located in proximity to the nuclear envelope and nucleolus through interactions with Xist and Firre. Most commonly, miRNAs undergo structural maturation and then participate in silencing other RNA sequences. Some other regulatory RNAs modulate the expression of cytoplasmic proteins that play a role in signaling.

The versatile physiological functions of regulatory RNAs are dependent on their subcellular distribution (Fig. 2). Generally, ncRNAs are largely localized in subcellular compartments based on their molecular phenotypes, thus actively contributing to cellular homeostasis. Native transcripts of ncRNAs, after maturation, are located in the nucleus or cytoplasm, with a relatively greater percentage in the nucleus than in the cytoplasm^79,80^. Posttranscriptional regulation directs the proper localization of most transcripts via RNA *cis*-acting elements and their corresponding functions^81^.

The nucleus is divided into subcompartments, including chromatin territories, the nucleolus, nuclear speckles, paraspeckles, and Cajal bodies, which are not membrane-enclosed. As a result, regulatory RNAs are largely located in the nucleus. Localization in these subnuclear compartments enables regulatory RNAs to participate in the machinery, acting in diverse aspects of cellular processes.

Chromatin has an intricate architecture and largely serves as the biological control tower for various cellular processes, including transcriptional regulation, DNA replication, and the cell cycle. In its protein-dense assembly within the genome structure, its tightly regulated hierarchical folding ensures spatiotemporally proper gene expression^82^. Among the myriad of cues that can result in chromatin alterations, regulatory RNAs are emerging as accessible mediators for interactive cooperation. These regulatory RNAs dynamically interact with genomic loci in response to the demands of numerous cellular processes. Chromatin-associated RNAs predominantly act at *cis*-regulatory sites to promote gene expression, as revealed by RNA-chromatin sequencing analysis, providing a global view of proximally driven interactions in cells and showing a propensity for an increased repertoire of chromatin-associated RNAs^83,84^. Although the precise mechanisms involving regulatory RNAs remain unclear, accumulating research indicates that many RNAs, especially lncRNAs, are involved in mechanisms of chromatin modulation. For instance, the long intergenic ncRNA *HOXA* transcript at the distal tip (*HOTTIP*) is generated from the 5′ end of the HOXA locus and has been suggested to regulate gene expression at distal sites. The *HOTTIP* RNA specifically interacts with chromatin via chromosomal looping. Suppression of *HOTTIP* RNA expression abolishes the dense occupation of the WDR5/MLL protein complex on the 5′ *HOXA* cluster, whereas properly transcribed *HOTTIP* facilitates the activation of transcription and H3K4 methylation via mutual interdependence on WDR5^85^. The *HOTTIP* RNA also mediates chromatin remodeling, targeting neighboring genes, including *HOXA9*, *FAS*, and *miR196b*, with a confirmed relationship to acute myeloid leukemia^86^. Another HOX cluster-related long intergenic ncRNA, HOX transcript antisense RNA (*HOTAIR*), whose sequence is located specifically within the HOXC sequence on chromosome 12, is known to regulate the chromatin state^87^. The *HOTAIR* RNA epigenetically silences gene expression by interacting with the lysine (K)-specific demethylase 1 (LSD1)/PRC2 protein complex located at promoter regions, leading to H3K4 and H3K27 methylations. The chromatin-modifying complex is supported by *HOTAIR* RNA as a scaffold to support its recruitment to sites near the chromatin of target genes and is composed of EZH2, EED, SUZ12, and RbAp46/48^88^. Moreover, PRC2 was revealed to be dispensable for the repression of the expression of certain genes by the *HOTAIR* RNA^89^. Several X chromosome-embedded regulatory RNAs, such as X-inactivation specific transcript (*Xist*) and Firre intergenic repeating RNA element (*Firre*), which are lncRNAs that are X-linked and participate in maintaining nuclear organization, have been shown to be restricted to the nucleus. *Xist* is a conserved and well-characterized regulatory RNA in terms of X chromosome inactivation (Xi) that performs multiple intranuclear processes through chromosome-wide regulation. *Xist* triggers gene silencing by coating the X chromosome at proximal sites and subsequently represses gene expression. The localization of Xi is also strongly related to *Xist*-associated assembly. The concomitant direct interaction of lamin B receptor (LBR) recruits Xi to the nuclear periphery, where the adjacent nucleolus appears to facilitate higher-order Xi loop formation and maintain heterochromatin^90–93^. In addition, other regulatory RNAs that modulate *Xist* RNA expression have been identified. One antisense RNA termed *Tsix* is required for homeostatic *Xist* expression, which prevents aberrant transcriptional activity of the X chromosome; on the other hand, *XistAR*, *Xert*, *Jpx*, and *Ftx* have been demonstrated to promote *Xist* expression in *cis*^92,94–96^. *Firre* is anchored on the inactivated X chromosome, cooperatively organizing intranuclear positioning with *Xist*. The nucleolar association and H3K27me3 modification suggest the localizing ability of *Firre*, although the relevant mechanisms are not fully understood^92,97^. In the context of regulatory RNA involvement in immune cells, myeloid RNA regulator of Bim-induced death (*Morrbid* RNA, initially identified as *Gm14005*) has been suggested to regulate *Bcl2lII* (a proapoptotic gene) at the transcriptional level. Cytokine-mediated *Morrbid* RNA expression induces the suppression of *Bcl2lII* in *cis* of the bivalent promoter, regulating the repressive histone mark H3K27me3 and EZH2 methyltransferase activity^98^.

The transient hybridization of RNA:DNA results in the formation of R-loops, which displace single-stranded DNA, an essential structure for genome-wide regulation, particularly the regulation of chromatin dynamics and transcription. Enriched R-loops can be observed at active genes across the genome, posing a threat to a stable DNA topology^99^. Abnormally established and accumulated R-loop structures can lead to stalled transcription and replication^100^. In addition to nascent RNAs that form R-loops, other classes of RNAs (such as regulatory RNAs) form or utilize this structure for gene expression through epigenetic regulation^101^. In mouse embryonic stem cells (mESCs), the lncRNA *Lnc530* was revealed to regulate R-loop formation to prevent excessive accumulation of two local proteins, TDP-43 and DDX5^102^. Another lncRNA, *TARID*, also in mESCs, binds to GADD45A, which is followed by the recruitment of DNA demethylases to promote *TCF21* transcription by the formation of R-loops that occupy the *TCF21* gene promoter^103^. In addition to lncRNA-mediated R-loop formation, R-loops themselves tether lncRNAs, recruiting several proteins with functions in regulating target genes.

The essential regions responsible for transcriptional regulation across noncoding regions of the genome, namely, enhancers and promoters, coordinate to drive the expression of specific genes^104^. In an already open chromatin template where histone octamers are removed, transcription factors that recognize a subset of factors to be incorporated can bind. Most promoters are located upstream of coding genes, while enhancers can be located up- or downstream of genes distally. Both adopt a *cis* configuration with respect to the genes they regulate. Enhancers can be located spatially proximal to promoters due to chromosomal looping and the accommodation of multiple proteins, including transcription factors, RNA polymerases, and architectural proteins that anchor and cooperate for regulatory signaling to induce gene expression^105^. In some cases, multiple discrete enhancers may contribute to the broad induction of a specific gene, such as *c-fos*, under temporal or tissue-specific control^106^.

Sequential control by enhancer regions due to insufficient activity of a general promoter determines whether the promoter is activated. Enhancer RNAs (eRNAs) are a distinct class of ncRNAs that are uni- or bidirectionally synthesized from active enhancers and are generally expressed at low levels^107^. These eRNAs were first identified in neurons and indeed were shown to regulate genes under conditions of neuronal stimulation in response to membrane depolarization^108^. Under these conditions, an activity-dependent induced eRNA is produced during the transcription process to sequester the negative elongation factor (NELF) complex from the cognate promoter, promoting gene expression. The eRNA specifically recognizes the RNA recognition motif (RRM) embedded in the NELF-E subunit, facilitating the release of RNA polymerase II pausing to resume elongation^109^ and stabilizing chromosomal looping via a cohesion complex^28,110^. The ability of eRNA–NELF binding to release NELF was further investigated and found to be dependent on the preference of NELF for guanosine nucleosides within sequences of more than 200 nt, which are bound by the multivalent tentacles of NELF^111^. The mechanism through which enhancers find their cognate promoters is largely unclear, recently, transposable sequence-mediated pairwise enhancer-promoter interactions throughout the genome have been suggested. Genome-wide mapping through RIC-seq has enabled us to understand the complementary interactions between RNA–RNA pairs and their potential mediator sequences. Enhancer-promoter selectivity has been suggested to be mediated by eRNA and antisense transcripts upstream of the promoter (i.e., uaRNAs), and 37.9% of the juxtaposing sequence in the eRNA-uaRNA interaction was found to be Alu elements^112^. Super-enhancer regions have been identified and assumed to determine cell identity via multiple clustered enhancers^113,114^. The eRNAs inferred to promote enhancer activity were repeatedly expressed at the highest levels when controlled by superenhancers, which may govern tumor heterogeneity^115^. With respect to the cooperation of enhancers and promoters, the 7SK snRNA is considered an essential regulator of transcription that forms a complex with positive transcription elongation factor b^116^. They were demonstrated to promote transcription in two ways, via mRNA or RNA polymerase II-specific snRNAs, and are involved in the repressive modulation of eRNAs^117,118^.

After nuclear export, mRNAs undergo translation, and the miRNA biogenesis pathway similarly progresses from the nucleus to the cytoplasm. Although miRNAs are more abundant in the cytoplasm than in the nucleus, nuclear-resident miRNAs have recently been recognized for their noncanonical roles^119,120^. Several lines of evidence suggest that miRNAs function in the nucleus through the nucleocytoplasmic transport of the RNA-RISC complex. The RISC cleaves transcripts, suggesting that miRNAs play a role in guiding this complex to cognate regions, even on RNAs of other classes^121–123^. Outside the nucleus, exported mRNA sequences undergo further processing for translation into proteins. miRNAs are known to regulate posttranscriptional gene expression through recognition of base pairs, particularly in the 3′-UTRs of mRNAs in the cytoplasm. This event has historically been considered an important negative regulator of mRNA stability and translation^18^. The gene-silencing machinery directed by miRNAs can influence any mechanistic layer tuned for operation by the proper abundance of a protein. A nearly perfect complementary match (seed sequence) of 2–7 nucleotides targets a transcript, leading to its degradation and translational repression. Numerous types of miRNAs have been identified and revealed to be functionally involved in a myriad of cellular processes. For example, miR-10a-3p regulates *KLF3-AS1* and *ZBTB20* to promote apoptosis and extracellular matrix synthesis^124^, and miR-182-3p targets *TRF2* to promote telomeric integrity^125^. One of the sophisticated miRNAs is miR-21, which targets *PDCD4*, *TPM1*, and *PTEN* for tumor suppression and is involved in diverse processes in cellular activities^126,127^. Notably, miRNAs can be sequestered by other competitive endogenous RNAs that share complementary sequences, ensuring the regulation of translation^128^. However, many roles of miRNAs in certain physiological processes have not yet been fully elucidated through identification of their cognate partners or cooperative molecules.

Cell signaling, as a fundamental communication ability of cells, is initiated and maintained by associated molecular cascades. Similarly, lncRNAs are crucial regulators in the cytoplasm, where they participate in intracellular cascades^129,130^. The lncRNA steroid receptor RNA activator-like noncoding RNA 1 (*SLNCR1*) interacts with the N-terminus of the androgen receptor to regulate downstream pathways. The *SLNCR1* RNA occupies the androgen receptor in a sequence-dependent manner, activating a proinvasion signaling cascade in melanoma^131^. Cardiac apoptosis-related lncRNA (Carlr) preferentially interacts with p65 before its nuclear translocation, which regulates NF-κB signaling-associated genes^132^. PRR34 antisense RNA 1 (*PRR34-AS1*) was shown to regulate the Wnt/β-catenin pathway by sequestering miRNA-296-5p, which regulates E2F2 and SOX12^133^. The extracellular environment is another location where regulatory RNAs function. The dysfunctional telomere-derived shortened form of telomeric repeat-containing RNA was found to be secreted in exosomes, thus transmitting cytokine signals to other cells^134^. Beyond the cellular localization of regulatory RNAs, spatial transcripts across tissues also perform distinct functions according to the spatial context.

The highly discrete compartment-dependent distribution and constellation of regulatory RNAs need to be finely tuned to subsequently control the molecular machinery to maintain cellular homeostasis. Consequently, many undefined aspects of regulatory RNA-associated phenomena need to be delineated.

## Regulatory RNAs involved in epitranscriptomic modifications

**Fig. 3:**
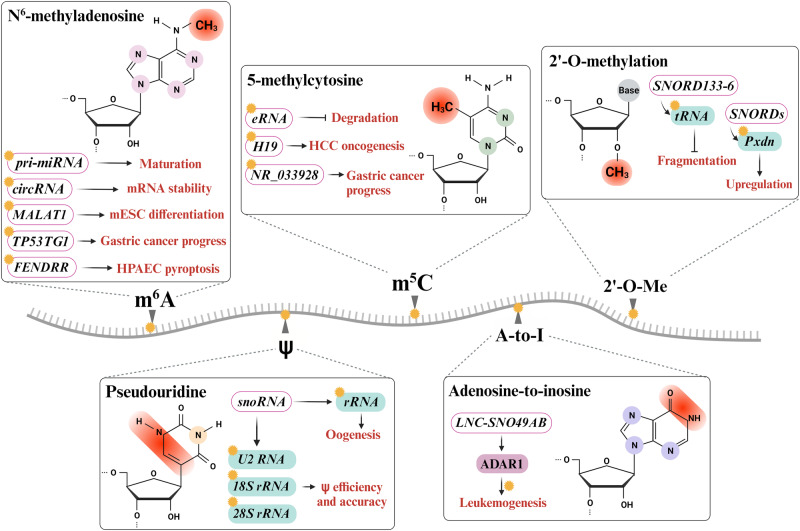
RNA modification-induced epitranscriptomic modification of regulatory RNAs. Many RNA modifications, including m^6^A, m^5^C, 2′-O-Me, A-to-I, and ψ, have been identified in various RNAs. Multiple epitranscriptomic RNA modifications regulate and modulate cellular functions such as miRNA maturation, mRNA stability, gene expression, translation efficiency, degradation, and ESC differentiation. Additionally, these modifications are associated with various pathophysiological conditions, including gastric cancer, HCC, and leukemogenesis.

DNA and histone modifications are common factors mediating epigenetic regulation^135–137^. In recent research, posttranscriptional RNA modifications such as N^6^-methyladenosine (m^6^A), 5-methylcytosine (m^5^C), N^1^-methyladenosine (m^1^A), 2′-O-Me, adenosine-to-inosine (A-to-I) editing, and ψ have been comprehensively investigated. These are known as epitranscriptomic regulators that play important roles in regulating gene expression and various cellular processes. The reciprocal impact of RNA modifications on regulatory RNAs underscores the intricate interplay between epitranscriptomic modifications and regulatory RNA-mediated processes^138,139^ (Fig. 3). Therefore, the importance of RNA modifications in epitranscriptomic regulation is increasingly recognized as a crucial aspect of cellular function^140,141^.

### N^6^-methyladenosine (m^6^A)

m^6^A, methylation of the adenosine at the N^6^ position, is the most characterized and abundant internal RNA modification. This modification has been identified in coding and noncoding RNAs, such as mRNAs, tRNAs, rRNAs, lncRNAs, miRNAs and eRNAs. In particular, it is predominantly found in 5′- and 3′-UTRs, long internal exons, and near stop codons and is associated with the RRACH (R = A or G, H = A, C, or U) consensus sequence. This modification is catalyzed by “writer” complexes, such as the methyltransferase 3 (METTL3), METTL5, METTL14, METTL16, Wilms tumor 1-associated protein, RNA binding motif 5, and zinc finger CCCH-like domain-containing protein 13 complexes. These m^6^A methyltransferase complexes participate in the process of target RNA modification^142–144^. The removal of m^6^A is orchestrated by several “erasers”, including fat mass and obesity-associated protein and human AlkB homolog H5 (ALKBH5) with α-ketoglutarate. The “readers” of m^6^A, such as YTH domain-containing family (YTHDF), YTH domain-containing 2 and insulin-like growth factor 2 mRNA-binding protein 1, recognize m^6^A at different positions, thereby regulating biological functions involved in transcription, alternative splicing, RNA decay, RNA stability, nuclear export, and translation^145–147^. For instance, pri-mRNAs undergo several cleavage steps in the conversion from precursor miRNAs (pre-miRNAs) to mature miRNAs during miRNA biogenesis, which is regulated by various factors, including RNA methylation. Pri-miRNAs have enrichment of an m^6^A motif (GGAC), and it can undergo m^6^A modification by METTL3, which is recognized by heterogeneous nuclear ribonucleoprotein A2/B1 and causes miRNA processing via DiGeorge syndrome critical region 8. Thus, the level of a mature miRNA is modulated by the level of m^6^A deposited via METTL3^148,149^. Similar to mRNAs, circRNAs have numerous m^6^A modifications^150^. m^6^A-induced circRNAs participate in protein synthesis via the interaction of YTHDF3 and eIF4G2, which is an initiation factor. In addition, recognition of m^6^A-modified circRNAs by YTHDF2 is reported to be the key element in RNA degradation, and it also controls the stability of mRNAs and circRNA-associated immunity^151,152^. Previous studies have reported that m^6^A-modified lncRNAs regulate RNA‒protein and RNA‒RNA interactions, such as the binding of heterogeneous nuclear ribonucleoprotein C to the U5 tract of metastasis-associated lung adenocarcinoma transcript 1 (*MALAT1*) lncRNA and the interactions of large intergenic coding RNA 1281 (*linc1281*) with the let-7 family of miRNAs, which are associated with mESC differentiation^153,154^. In addition, aberrant m^6^A regulation is significantly associated with diverse types of human diseases, including cancers, brain diseases, and cardiovascular diseases^155–157^. The level of TP53 target gene 1 (*TP53TG1*) lncRNA, a critical tumor suppressor, is decreased in gastric cancer. *TP53TG1* has abundant m^6^A modification sites, but the demethylase ALKBH5 abolishes *TP53TG1* stability, reduces its expression level and promotes its degradation, and suppresses the PI3K/AKT pathway by binding cellular inhibitor of protein phosphatase 2A, resulting in the inhibition of cell cycle progression and proliferation in gastric cancer. Thus, m^6^A-mediated *TP53TG1* downregulation is closely related to the progression of gastric cancer^158^. The level of the lncRNA fetal-lethal noncoding developmental regulatory RNA (*FENDRR*) is markedly reduced in human pulmonary artery endothelial cells (HPAECs), which leads to hypoxia-induced HPAEC pyroptosis. The m^6^A “reader” YTHDC1 recognizes m^6^A-modified *FENDRR* and induces its degradation. m^6^A-modified *FENDRR* interacts with the dynamin-related protein 1 (DRP1) promoter by forming an RNA‒DNA triplex that mediates its methylation and alters its transcription; therefore, DRP1 expression is low in healthy individuals. However, the m^6^A-mediated decay of *FENDRR* via interaction with YTHDC1 increases DRP1 expression, contributing to HPAEC pyroptosis^159^.

### 5-methylcytosine (m^5^C)

m^5^C, methylation of the fifth carbon of the RNA base in cytosine (C), is another common RNA modification. To date, m^5^C has been detected in a wide range of RNAs, including mRNAs, tRNAs, rRNAs, and eRNAs. Among these RNAs, m^5^C is abundant in eukaryotic tRNA and rRNA^160,161^. Members of the DNA methyltransferase-like 2 and NOL1/NOP2/SUN domain family member (NSUN) families mediate m^5^C modification in specific types of RNA. In addition, members of the ten-eleven translocation family function as m^5^C demethylases, while Aly/REF export factor and Y-box binding protein 1 act as readers to recognize m^5^C by binding to m^5^C-modified sites^162,163^. m^5^C modification contributes to multiple cellular functions that differ by RNA subtype^140,163^. m^5^C modification of eRNA prevents its degradation^164^. Moreover, m^5^C modification is closely related to the progression of various types of cancer^160,165,166^. The *H19* lncRNA is aberrantly overexpressed and is known to have carcinogenic effects as a tumor-associated factor. In hepatocellular carcinoma (HCC), both the *H19* lncRNA and m^5^C methylation are increased, which is significantly related to m^5^C-induced *H19* lncRNA expression and is uniquely connected to the Ras-GTPase-activating protein SH3 domain-binding protein 1 (G3BP1). G3BP1, an oncoprotein, can bind to *MYC* mRNA, which causes its decay and promotes tumor progression. The increased level of m^5^C-modified *H19* lncRNA can compete with G3BP1, which suppresses the degradation of *MYC* mRNA and induces an increase in the MYC level, thus enhancing the oncogenic process^167^. In gastric cancer, the lncRNA *NR_033928* is upregulated and highly m^5^C methylated by overexpressed NSUN2, which contributes to poor prognosis. m^5^C-modified *NR_033928* was found to promote an increase in glutaminase, which led to an increase in α-ketoglutarate (α-KG), a glutamine metabolite, and α-KG upregulated the expression of *NR_033928* by activating 5-hydroxymethylcytosine demethylation, which promoted the progression of gastric cancer^168^.

### 2′-O-methylation (2′-O-Me)

2′-O-Me, a highly conserved posttranscriptional modification of RNA, refers to the attachment of a methyl group to the 2′ hydroxyl (–OH) of the ribose moiety in any nucleotide via SNORDs and the 2′-O-methyltransferase fibrillarin. This modification has been identified in a wide range of RNAs, including mRNAs, tRNAs, rRNAs, and snRNAs, which play important roles in various cellular processes. For instance, SNORDs and fibrillarin-mediated 2′-O-Me in peroxidasin *(Pxdn)* promote the upregulation of *Pxdn* mRNA expression^169^. In the human ribosome, 2′-O-methylation of rRNA can be regulated at functional sites important for translation, and the intrinsic capabilities of the ribosome modulate the translation of mRNA by altering the 2′-O-Me pattern^170^. In addition, *SNORD113-6*-mediated 2′-O-Me modification of tRNA^Ler^(TAA) inhibits the site-dependent fragmentation of a tRNA fragment (tRF)^Leu^, which is associated with vascular remodeling^171^. Moreover, 2′-O-Me modification is correlated with lncRNA modulation. The *ZFAS1* lncRNA and snoRNP NOP58 are upregulated in colorectal cancer (CRC), and *ZFAS1* interacts with NOP58, which results in the binding of *SNORD72C* and *SNORD78* to mediate 2′-O-Me at the *Gm3878* and *Gm4593* sites in rRNA. As a result, the mRNA stability and translation of the downstream targets eukaryotic translation initiation factor 4A3 and laminin subunit gamma 2 increase, which promotes CRC cell proliferation and progression^172^. However, not all SNORDs do not participate in methylation. In HCC, lncRNA associated with liver regeneration (*LALR1*) and *SNORD72* are highly upregulated, and *LALR1* binds to *SNORD72*, which promotes the stability of inhibitor of DNA binding 2 mRNA. Subsequently, it enhances invasion and tumor growth^173^.

### Adenosine-to-inosine (A‒I) editing

A-to-I editing is the conversion of adenosine to inosine, via the deamination of the targeted adenosine to inosine. This reaction is catalyzed by adenosine deaminase acting on RNAs (ADARs), of which there are three, i.e., ADAR1, ADAR2, and ADAR3, but ADAR3 lacks catalytic activity^174,175^. The snoRNA-related lncRNA *LNC-SNO49AB* has two C/D box snoRNA sequences, *SNORD49A* and *SNORD49B*, which also contain a 5 m^7^G cap and a 3′ snoRNA structure. It is highly upregulated in leukemia and promotes not 2′-O-Me modification but A-to-I editing by binding to ADAR1, which promotes hematopoietic malignancy^176^.

### Pseudouridine (ψ)

ψ, the first confirmed RNA modification, identified in 1951, is an isomer of the nucleoside uridine that is composed of a carbon‒carbon (C1–C5) glycosidic bond instead of a nitrogen-carbon (C1-N1) glycosidic bond. ψ is significantly conserved across species and is found in most cellular RNAs, including mRNAs, tRNAs, rRNAs, and snRNAs. This modification is mediated by pseudouridine synthases, whose unique cellular localization and specific RNA targets have been reported^177,178^. Additionally, several unique targets need the participation of the RNA‒protein complex H/ACA box RNP, which is composed of four distinct proteins and a guide RNA and localized in the nucleolus and nucleoplasmic Cajal bodies. snoRNAs, as small nuclear guide RNAs, designate the regions of ψ modifications mediated by H/ACA box RNPs^179,180^. The intronic H/ACA snoRNA leads to pseudouridylation in the large rRNA subunit at positions 2258 and 2260 and is termed snR191 in yeast and hU19 in humans; the highly conserved ψ modification is beneficial for the cell^181^. Specifically, in oocytes, ψ modification of rRNA through the H/ACA box snRNP complex is required during oogenesis^182^. Furthermore, H/ACA box guide RNAs are composed of two hairpins that carry the internal ψ guide loops that allow site-dependent ψ of spliceosomal and ribosomal RNAs in humans. A recent study reported four human H/ACA RNAs (*SNORA53*, *SNORA57*, *scaRNA8*, and *scaRNA1*) that transfer ψ loops to promote the maintenance of two specific ψ modifications on U2 (Ψ43/Ψ44 and Ψ89/Ψ91) RNA, 18S (Ψ1045/Ψ1046), and 28S (Ψ3747/Ψ3749). These ψ loops have advantages in terms of efficiency and accuracy because of the versatility of ψ^183^.

Epitranscriptomic regulation via RNA modifications has been gradually investigated for its unique functions in various cellular processes and human diseases through advanced sequencing techniques. Novel RNA modifications in regulatory RNAs have continually been reported, and this evidence has contributed to the clarification of biological functions of RNAs. However, there are still numerous conflicting factors demonstrating the biochemical properties of RNAs inside and outside the cell; thus, additional studies are needed to determine the versatility of RNA modifications.

## Interactions of regulatory RNAs with RNA-binding proteins

**Fig. 4:**
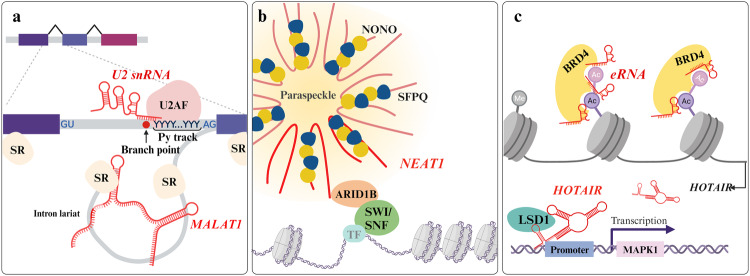
Cooperation between regulatory RNAs and RNA-binding proteins. **a** U2 snRNA participates in the splicing process by recognizing the branch point, a process assisted by U2AF on the Py track near the 3’ splice site. MALAT1 and SR proteins form intron lariats for alternative splicing. **b** NEAT1 is a well-defined scaffolding lncRNA localized in paraspeckles in the nucleus that can sequester proteins such as NONO and SFPQ. NEAT1 also mediates chromatin remodeling by binding to ARID1B. **c** BRD4 recognizes acetylated K residues in a manner mediated by eRNAs and regulates HOTAIR expression levels. HOTAIR facilitates transcriptional regulation via LSD1 recruitment.

Regulatory RNAs themselves can be functional units; however, they mostly form dynamic RNA‒protein partnerships and function as central mediators or in mutual support. These interactions involve the binding of RNA to proteins and can occur reciprocally, with proteins recognizing RNA specifically through various RNA-binding domains such as the RRM, double-stranded RNA binding motif (dsRBM), DEAD/DEAH-box helicase domain, K homology (KH) domain, and zinc finger. Notably, recent studies have expanded our understanding of RNA‒protein interactions beyond conventional binding domain-mediated modules^184^. In this context, the functional bridge constructed by interactions between RNA and proteins plays intricate biological roles, contributing to the complexity of the cellular regulatory network (Fig. 4).

Prior to the nuclear export of synthesized RNA transcripts, primary transcripts undergo an RNA processing program that is elaborately orchestrated by numerous macromolecules. This processing program includes splicing and maturation, resulting in the establishment of a highly diversified transcriptome and proteome. A prominent example of higher-order assembly is the spliceosome, a multimegadalton macromolecular apparatus that participates in and regulates splicing tightly in direct and indirect ways and involves the participation of regulatory RNAs^185^. Nuclear speckles, located in the interchromatin space, provide platforms for preparing the splicing of diverse classes of RNAs^186^. The representative splicing process begins when the spliceosome binds to the splice sites of heterogeneous nuclear RNAs (hnRNAs), forming a complex referred to as an snRNP that is composed of snRNAs and confers recognition ability and catalytic activity. This module mediates transesterification and sequence rearrangement^187^. The U2 snRNA is a major snRNA that facilitates the recognition of the intronic branch point via nucleotide base pairing, initiating recognition of the 3′ splice site. The recognition of the 3′ splice site is facilitated by U2 auxiliary factor (U2AF) due to branch point degeneracy^188,189^. The heterodimeric U2AF complex (containing 65 and 35 kDa subunits) specifically detects the polypyrimidine (Py) tract element upstream of the 3′ splice site and then accomplishes spliceosome assembly followed by lariat formation for the excision of the designated intron sequence^190^. Nuclear speckles for the assembly of the large spliceosome are constructed via the contribution of lncRNAs. *MALAT1*, a highly expressed lncRNA, has been demonstrated to recruit serine- and arginine-rich (SR) proteins that dock to pre-mRNA sequences, thereby regulating splicing^191^. *MALAT1* not only interacts with SR splicing factors but also governs phosphorylation levels, subsequently influencing alternative splicing events^192^. Two splicing factor proteins, Py tract-binding protein 1 and PTB-associated splicing factor (PSF), are implicated as other regulatory coordinators in splicing, interacting with *MALAT1*. PTBP1 and PSF become stabilized through direct interaction with *MALAT1*, resulting in the exclusion or inclusion of exons during alternative splicing^193^. Splicing and maturation also occur in other classes of RNAs through component regulatory RNAs and RNPs: in ribosomal RNAs in the nucleolus via snoRNAs, in snRNAs in Cajal bodies via scaRNAs, and in histone pre-mRNA in histone locus bodies via snRNAs during processing^194^. These regions are transcriptionally active and are referred to as membraneless nuclear bodies.

As nuclear bodies, paraspeckles are sphere-like RNP granule structures that utilize *NEAT1* lncRNA as an assembly scaffold. These structures are commonly found in proximity to nuclear speckles. Although substantial information is needed for a full understanding, paraspeckles are suggested to function as platforms that segregate certain RNAs or proteins, resulting in molecular functions associated with the sequestered molecules^195^. The splicing factor proline/glutamine-rich (SFPQ) plays versatile roles by acting as a splicing factor and transcriptional regulator and is also a core component in paraspeckle retention through direct interaction with *NEAT1*. Increased paraspeckle formation is triggered under stressful conditions, such as intracellular invasion. This may sequester SFPQ, which is a suppressor of interleukin-8 cytokine transcription^196^ and a transcription factor of the *ADARB2* gene^197^. Non-POU domain-containing octamer-binding protein (NONO) is another essential component that contributes to the coalescence of paraspeckles and regulates gene expression along with SFPQ. Additionally, NONO participates in the DNA double-strand break repair mechanism through recruitment of another protein, RPLP0. X-irradiation-induced DNA damage promotes NONO condensation in *NEAT1*-associated paraspeckles, and NONO then interacts with RPLP0, leading to nonhomologous end joining repair^198^. Moreover, recent findings have revealed the direct implication of *NEAT1* in interacting with paraspeckles and the chromatin remodeling complex. ATP hydrolysis allows the repositioning of nucleosomes via binding to the switch/sucrose nonfermentable (SWI/SNF) complex, which increases the accessibility of other molecules for transcriptional regulation^199^. Coordination between SWI/SNF and paraspeckles by a specific interactor, ARID1B, has been demonstrated, and this interaction allows the recruitment of various kinds of chromatin-associated proteins to influence gene regulatory processes, leading to alternative splicing. This interaction was markedly detected only with the canonical BAF (cBAF) among the three distinct mammalian SWI/SNF complexes, and cBAF is known to govern the enhancer region^200,201^.

The nucleosome positioning process, which involves sliding or dissociating DNA for gene regulatory mechanisms, is typically referred to as chromatin remodeling. The flexibility of chromatin arises from its rearrangement through an ATP-dependent mechanism to open DNA grooves. The highly conserved bromodomain (BRD) recognizes acetylations at K residues on histone tails and induces the assembly of BRD-containing proteins primarily for transcriptional regulation^202^. The BRD4 protein contributes to super-enhancer organization, with tandem BRDs utilizing the eRNA-BRD4 interaction to promote tethering to acetylated K residues^203^. Alongside the conserved transcriptional regulatory elements for lncRNAs, BRD4 was found to have a direct interaction with the *HOTAIR* promoter, controlling the *HOTAIR* transcript level^204^. Regarding the role of *HOTAIR* after BRD4-mediated promotion, *HOTAIR* can attract LSD1 to decrease the H3K9me2 level of the *MAPK1* promoter^205^. Several related lncRNAs, namely, *H19*, *HOTAIRM1*, *DGCR5*, and *MEG3*, were also revealed to be related to BRD4-associated regulation^204^. Furthermore, BRD4 plays a functional role after forming a complex with *NEAT1*, which is negatively regulated via alterations in WDR5 or EZH2 activity^206^. A recently identified lncRNA, *LENGA*, acts as a connector, enhancing the interaction between the transcription factor TP53 and BRD7. BRD7, a subunit of the polybromo-associated BAF SWI/SNF complex, uses regulatory RNAs as a scaffold to regulate downstream gene expression^207^. The core subunit SMARCB1 of SWI/SNF and its suggested partner, *SMARCB1* lncRNA, together modulate the expression of the GAS6 oncogene^208^. The catalytic subunit BRG1 of the SWI/SNF complex interacts with the *uc.291* lncRNA derived from ACTL6A to regulate the transcription of EDC genes^209^. As described above, based on these remarkably sophisticated intracellular contributions of regulatory RNAs, intercellular communication and a series of changes can be explained by the interdependence of proteins and regulatory RNAs.

## Conclusions

In the rapidly evolving field of regulatory RNAs, novel and functional RNAs have been consistently uncovered, paving the way for the development of meaningful RNA-centric technologies based on modern biology. Notably, recent global efforts to combat coronavirus disease 2019 have accelerated the development of mRNA vaccines, which have shown remarkable potential as therapeutic countermeasures^210–212^. Within this context, various RNA-based therapeutics, including small interfering RNAs, antisense oligonucleotides (ASOs), CRISPR/Cas9 gene editing, anti-miRs (or antagomirs), aptamers, miRNA sponges, therapeutic circRNAs and mRNA vaccines, have emerged to address diverse pathologies. These therapeutics modulate the expression of mRNAs as well as ncRNAs by targeting cognate sequences^213,214^ and have been focused primarily on well-defined mRNA targets^215–219^. For instance, ASOs have been employed in the treatment of Duchenne muscular dystrophy, with casimersen gaining approval for its modulatory effects on splicing in intergenic regions^220^. Antagomirs, antisense miRNAs, exhibit nuclease resistance through 2′-methoxy substitution. The results of clinical trials of MRG-110, targeting miR-92a, in angiogenesis and CDR132L, targeting miR-132, in heart failure (phase I: NCT03603431 and phase Ib: NCT03494712) underscore the therapeutic potential of antagomirs^221^. RNAs have a strong negative charge, which renders them plasma membrane-impermeable and susceptible to degradation by RNases, and thus their ability to attain physical proximity to their precise target sequences is limited, and efforts to overcome these challenges are ongoing^219^. Numerous studies are underway to overcome these limitations by exploring modifications to the phosphodiester backbone and employing viral-vector-based and nonviral delivery systems^220,222^, nanocarriers such as lipid nanoparticles, cationic polymer-based polyplexes, spherical nucleic acids, DNA nanostructures and exosomes^219^. For instance, the GalNAc-conjugated antagomir AZD4076 (RG-125), designed to improve the delivery of targeting ligands, was developed to target intracellular miR-103/107 but was discontinued in phase I trials (NCT02612662 and NCT02826525)^214^. Antagomirs, on the other hand, are strategically designed to exhibit increased permeability via conjugation to cholesterol^223^.

The continuous investigation of applicable regulatory RNAs and their effectiveness of delivery remains crucial for understanding their biological roles in response to a wide range of cellular and environmental conditions. As the field progresses, the exploration of innovative therapeutic approaches and delivery strategies holds promise for addressing the complex challenges associated with RNA-based interventions.

Many studies have attempted to predict the biological functions of regulatory RNAs, recognizing their substantial potential as key cellular regulatory factors^224–226^. However, defining only single time point-dependent conformations and versatilities of important RNAs is insufficient, as intrinsic and extrinsic factors differ between physiological and disease conditions. The important biochemical properties of RNAs, including their folding stability, binding affinity, specificity, modification sites, and catalytic efficiency, still cannot be predicted by sequencing-based quantitative measurements^61,138,227,228^. Undoubtedly, deep learning approaches have become important for expanding strategic analyses to apply RNA-related predictions using various sequencing-based datasets^229–233^. The data-driven approach excels at extracting optimal patterns and knowledge through sophisticated computational processes, leveraging massive training data and convolution operations in multiple hidden layers within various neural network algorithms^234–236^. Recent studies have reported a wide range of RNA-related predictions using diverse deep learning methods, such as multiple types RNA modification sites predictor, ensemble multiscale deep learning predictor, Deepm5C, PseUdeep, Deep-2′-O-Me, and DLm6Am for predicting modifications^234,237–241^; DeepMirTar, deep donor splice site recognizer, and RBPsuite for predicting specific target sites^242–244^; DeepLncLoc for predicting localization^245^; ncRDense and circDeep for predicting classification^246,247^; UFold, NuFold, and deep learning-ALIgned nucleic acids for predicting structure^248–250^; and rBPDL and DeepBtoD for predicting RNA-binding proteins^251,252^ (Table 2). However, the practical correlations among these results needs to be demonstrated for more accurate predictions because in deep learning approaches, information is acquired from the input data; therefore, batch variability may influence the output data. The accuracy of predictive analyses with genomic data-based deep learning seems to be significantly dependent on the quality and quantity of the training data. Therefore, deep learning requires more refined and integrated training data obtained from various studies. Therefore, precise and highly efficient results that previously could not be obtained via biochemical experiments can now be obtained. Accelerated research in this field employing cutting-edge techniques is expected to expand to establish strategic approaches for RNA-targeting therapies based on a comprehensive understanding of RNA regulatory mechanisms.

**Table 2:** The current applied deep learning approaches and algorithms for the prediction of a wide range of RNAs.

Deep learning method	Application	Prediction Type	Algorithm	Ref.
DeepMRMP	Modification	m^1^A, Ψ, and m^5^C	Recurrent neural network (RNN)	^234^
EMDLP		m^1^A and m^6^A	Convolutional neural network (CNN) and long short-term memory (LSTM)	^239^
DLm6Am		m^6^Am	CNN and LSTM	^241^
Deepm5C		m^5^C	CNN and LSTM	^240^
PseUdeep		Ψ	CNN	^238^
Deep-2’-O-Me		2’-O-Me	CNN	^237^
DeepMirTar	Specific target site	miRNA target site	Stacked denoising autoencoders (SdA) in a deep belief network (DBN)	^242^
DeepDSSR		Donor splice site	CNN and LSTM	^243^
RBPsuite		RNA‒protein binding site	CNN and LSTM	^244^
DeepLncLoc	Localization	lncRNA subcellular localization	CNN	^245^
UFold	Structure	Secondary RNA structure	Fully convolutional network (FCN)	^248^
AliNA			U-Net convolutional network in CNN	^250^
NuFold		Tertiary RNA structure	AlphaFold in CNN	^249^
rBPDL	RNA-binding proteins	-	CNN and LSTM	^251^
DeepBtoD		-	Multiscale CNN	^252^
ncRDense	Classification	Noncoding RNA	DenseNET in CNN	^247^
circDeep		Circular RNA	Asymmetric CNN and bidirectional LSTM	^246^

Owing to exponential advancements in modern biological techniques, we now have access to massive amounts of genome-wide information. Dissecting molecular layers, particularly within the RNA-associated cellular machinery, seems likely to provide a more comprehensive understanding. However, there are many hurdles to overcome in interpreting the functions of the myriad of regulatory RNAs, in accordance with their associated macromolecule complexes and distributions in both the nucleus and the cytoplasm. Regulatory RNAs are more prevalent than previously appreciated but exhibit lower sequence conservation and expression levels than coding RNAs. Addressing these challenges requires enormous effort and critical considerations, including exceeding the baseline of transcriptional noise due to technical limitations. Revealing the isoforms and stoichiometries of regulatory RNAs under various physiological conditions is crucial for obtaining a deeper understanding of these RNAs. Gaining such an understanding of the extensive properties of regulatory RNAs is expected to significantly increase insights into the intricacies of organisms.
